# Supplementary material for: eMZed 3: flexible and interactive development of scalable LC-MS/MS data analysis workflows in python
Source: Bioinform Adv. 2026 May 15;6(1):vbag138. doi: 10.1093/bioadv/vbag138 (PMC13242181; doi:10.1093/bioadv/vbag138)
Supplement: vbag138_Supplementary_Data [file vbag138_supplementary_data.pdf]

# Supplementary Material

## eMZed 3: flexible and interactive development of scalable LC-MS/MS data analysis workflows in Python

**Uwe Schmitt, Jethro L. Hemmann, Nicola Zamboni, Julia A. Vorholt, Patrick Kiefer**

Table of contents:

- Table S1

**Table S1.** Comparison of features of eMZed 3, XCMS 4 (the R package), and mzmine 4.

<table>
<tr><th></th><th></th><th>eMZed 3</th><th>XCMS 4</th><th>mzmine 4</th></tr>
<tr><td rowspan="8">General</td><td>Language</td><td>Python</td><td>R</td><td>Java</td></tr>
<tr><td>License</td><td>MIT</td><td>GPL</td><td>MIT</td></tr>
<tr><td>Programming required</td><td>✓</td><td>✓</td><td>x (GUI)</td></tr>
<tr><td>Automation</td><td>Python code</td><td>R code</td><td>XML batch files</td></tr>
<tr><td>Interactive visualization of raw data, peaks, and spectra</td><td>✓</td><td>x</td><td>✓</td></tr>
<tr><td>Headless processing</td><td colspan="3">✓</td></tr>
<tr><td>Out-of-memory backend</td><td colspan="3">✓</td></tr>
<tr><td>Operating system</td><td colspan="3">Windows, macOS, Linux</td></tr>
<tr><td rowspan="10">Core processing</td><td>Peak / feature detection</td><td>✓<br>(Available algorithms: OpenMS, MZmine, XCMS)</td><td>✓</td><td>✓</td></tr>
<tr><td>Retention time alignment</td><td colspan="3">✓</td></tr>
<tr><td>Mass (re-)calibration</td><td colspan="3">✓</td></tr>
<tr><td>Gap filling</td><td>x (via e.g. scikit-learn)</td><td>✓</td><td>✓</td></tr>
<tr><td>Peak / feature grouping</td><td colspan="3">✓</td></tr>
<tr><td>Isotope grouping</td><td colspan="3">✓</td></tr>
<tr><td>Adduct grouping</td><td colspan="3">✓</td></tr>
<tr><td>Peak normalization</td><td>x (via e.g. scikit-learn)</td><td>✓</td><td>✓</td></tr>
<tr><td>Targeted peak extraction / EIC integration</td><td colspan="3">✓</td></tr>
<tr><td rowspan="4">Data support</td><td>LC-MS</td><td colspan="3">✓</td></tr>
<tr><td>LC-MS/MS</td><td colspan="3">✓</td></tr>
<tr><td>Chromatogram data (e.g. MRM)</td><td>✓</td><td>✓</td><td>only with PRO subscription</td></tr>
<tr><td>Input formats</td><td>mzML, mzXML</td><td>mzML, mzXML, NetCDF</td><td>mzML, mzXM, NetCDF, vendor formats</td></tr>
<tr><td rowspan="3">Annotation</td><td>Spectral library matching</td><td>x (via export to other tools)</td><td>via R package MetaboAnnotation</td><td>✓</td></tr>
<tr><td>Formula prediction from exact mass</td><td colspan="3">✓</td></tr>
<tr><td>SIRIUS / CSI:FingerID integration</td><td>x (via export)</td><td>x (via export)</td><td>✓</td></tr>
<tr><td rowspan="2">Downstream analysis</td><td>Statistics and plots</td><td>via Python ecosystem and R ecosystem (emzed R-bridge)</td><td>via R ecosystem</td><td>partly built-in</td></tr>
<tr><td>Machine learning</td><td>via Python ecosystem</td><td>via R ecosystem</td><td>via export to other tools</td></tr>
</table>
